# Reviewing Evidence for Disturbance to Coral Reefs Increasing the Risk of Ciguatera

**DOI:** 10.3390/toxins17040195

**Published:** 2025-04-11

**Authors:** Michael J. Holmes, Richard J. Lewis

**Affiliations:** Institute for Molecular Bioscience, The University of Queensland, Brisbane 4072, Australia; m.holmes@imb.uq.edu.au

**Keywords:** ciguatera, ciguatoxin, *Gambierdiscus*, *Fukuyoa*, reef disturbance, surgeonfish, damselfish, parrotfish, grouper, coral trout, turf algae, Great Barrier Reef, coral reef, marine food chain

## Abstract

The hypothesis that disturbance to coral reefs creates new surfaces that increase the risk of ciguatera is premised upon the increased algal substrates that develop on these surfaces being colonised by high ciguatoxin (CTX)-producing *Gambierdiscus* species that proliferate and enter the ciguatera food chain. Current evidence indicates that new algal substrates are indeed rapidly colonised by *Gambierdiscus*. However, the requirement that these *Gambierdiscus* species include at least one that is a significant (high) CTX-producer is more likely a limiting step. While ambient environmental conditions impact the capacity of *Gambierdiscus* to bloom, factors that limit the growth of the bloom could influence (typically increase) the flux of CTX entering marine food chains. Additionally, new algal substrates on damaged reefs can be preferentially grazed to funnel ciguatoxins from *Gambierdiscus* to herbivores in disturbed reef areas. In societies consuming second trophic level species (herbivores, grazers, and detritivores), such funnelling of CTX would increase the risk of ciguatera, although such risk would be partially offset over time by growth (toxin-dilution) and depuration. Here, we review evidence for six potential mechanisms to increase ciguatera risk from disturbance to coral reefs and suggest a hypothesis where ecosystem changes could increase the flux of CTX to groupers through a shift in predation from predominately feeding on planktonic-feeding prey to mostly feeding on benthic-feeding prey, increasing the potential for CTX to accumulate. Evidence for this hypothesis is stronger for the Pacific and Indian Oceans, and it may not apply to the Caribbean Sea/Atlantic Ocean.

## 1. Introduction

Ciguatera is caused by eating normally edible, marine, warm-water fishes contaminated with ciguatoxins (CTX) [1]. It results from a chain of events that begins with the production of CTX by benthic dinoflagellates (*Gambierdiscus* spp. and possibly *Fukuyoa* spp.), which are then transferred through marine food chains to produce fish that cause human poisoning, with some analogs structurally bio-modified in the process [2,3,4,5,6,7,8]. Ciguatera in the Pacific basin appears to be caused mostly by two structural families of toxins that can co-occur in contaminated fish, analogs of Pacific-ciguatoxin-1 (P-CTX-1, also known as CTX1B) and analogs of P-CTX3C (CTX3C) [3,9,10]. The USFDA has set a conservative recommendation of 0.01 µg/kg CTX equivalents (eq.) for the safe consumption of seafood, whereas Japan has recommended a less stringent safety limit of 0.175 µg/kg [11,12]. The USFDA limit is based upon the analysis of Lehane and Lewis [13], suggesting 0.1 µg P-CTX-1/kg flesh may cause mild poisoning in 2 out of 10 people eating 500 g of flesh (i.e., consumption of 50 ng P-CTX-1 eq.). The less stringent safety limit in Japan may partially reflect smaller portion sizes of fish meals than those typically consumed in other cultures. Ciguatera in the Atlantic and Indian Oceans is mostly caused by different structural families of CTX, Caribbean-CTX (C-CTX) in the Atlantic Ocean and Indian Ocean-CTX (I-CTX) in the Indian Ocean [3,14,15,16,17,18,19,20].

A long-held hypothesis originating from the Pacific basin is that disturbance to coral reefs causes an increased risk of ciguatera [21,22,23,24,25,26,27,28,29,30,31,32,33,34]. This hypothesis was initially derived from observations of apparently increased ciguatera risk after damage to Pacific coral reefs during and in the aftermath of World War II and before the discovery of *Gambierdiscus* as an origin of CTX, with ciguatera being a problem for both Japanese and US forces in Micronesia during the conflict [35]. In what became known as the “new surface hypothesis”, Randall [23] suggested that disturbance created new surfaces for the causative organism to colonise and proliferate. This hypothesis implies that space for growth of *Gambierdiscus* populations is limiting or that post-disturbance food chains increase the transfer of CTX to species consumed by people. However, if new surfaces can increase the risk of ciguatera, then some natural phenomena, including violent storms, should also create conditions that promote ciguatera outbreaks [25,36]. Fleshy macroalgae and/or turf algae can colonise and dominate coral reefs after major disturbances [37,38,39,40,41,42,43,44,45], providing new surfaces for *Gambierdiscus* to attach and possibly proliferate. However, there are few direct studies that have tested this hypothesis. Kaly and Jones [46] could not find supporting evidence for the new surface hypothesis from studies on reef disturbance at sites in Tuvalu, and engineering works for the development of a small marina site on a fringing reef at Hayman Island on the Great Barrier Reef in the 1980s did not produce an increase in *Gambierdiscus* populations [4]. In the only study of the toxicity of surgeonfishes (*Ctenochaetus striatus*) from the Great Barrier Reef, Lewis et al. [47] found only low concentrations of CTX in *C. striatus* spearfished from John Brewer and Davies reefs, despite John Brewer reef being damaged by crown-of-thorns starfish (*Acanthaster* sp.) while the nearby Davies Reef was only lightly impacted at the time. Thus, if disturbance to coral reefs leads to increased ciguatera risk, food chain factors likely play a significant role, given that most disturbances do not appear to be associated with outbreaks of ciguatera.

Turf algae rapidly colonise new surfaces on coral reefs [45,48,49,50,51,52,53] that are, in turn, likely colonised by *Gambierdiscus* transferred through the water column. This probably happens quickly, given that *Gambierdiscus* can be detected within 24 hr of deployment of benthic screen assays [54,55]. Several authors have advocated a greater focus on turf algae as substrates for *Gambierdiscus* as part of food chains that contaminate carnivorous fishes that most often cause ciguatera [2,4,6,7,56,57,58]. This may be more relevant for the Pacific Ocean, where many more herbivorous fish feed on turf algae than macroalgae [59,60,61,62], with the possibility that turf algal ecosystems will expand as coral reefs continue to deteriorate in the Anthropocene [62,63,64,65]. However, the difficulty in sampling and quantifying benthic dinoflagellate populations from algal turfs [47,66,67,68] has limited research into the role of turf algae in ciguatera. This could be addressed if test screen assays [54,55] or other technologies [47,66,67,68,69] are more widely deployed to quantify benthic dinoflagellate populations on turf algae. Although turf algae are well recognized as part of the mix of inorganic and organic substrates that make up large parts of coral reefs, these habitats are often excluded from analyses of substrate dominance on reefs, biasing perceptions of what typical substrates are on coral reefs [62,63,70]. It is also unclear if high CTX-producing species of *Gambierdiscus* differentially distribute on different algal substrates. These factors confound the use of published substrate data to quantify the effects of disturbance to coral reefs on ciguatera risk. However, quantitation of *Gambierdiscus* concentrations on turf algae combined with remote sensing techniques that estimate the spatial extent of turf algae in shallow waters (<20 m) of coral reefs [71] could allow the quantification of reef substrates and potentially the development of predictive ciguatera risk assessment models for reefs [4,6,7].

Many coral reef systems, including the Great Barrier Reef, have been impacted by an increasing range and frequency of major disturbances from cyclones, outbreaks of crown-of-thorns starfish, increased catchment runoff from more intense rain events and ongoing development, and coral bleaching events due to increasing water temperatures associated with climate change [65,72,73]. At least for the Great Barrier Reef, this has not yet produced any obvious increase in outbreaks of ciguatera [4], which supports the contention that not all disturbances impact ciguatera risk. However, disturbances cause different impacts to coral reefs and act over different time scales. For example, intense marine heat waves can produce dead but intact coral skeletons, whereas tropical storms pulverize and remove coral structures to leave behind a more open planar surface, leading to different outcomes for algal growth and herbivory. Dead coral skeletons can not only facilitate the establishment of algae but, in some circumstances, favour an algal assemblage that is more resistant to control by herbivores [74]. Biological disturbances such as outbreaks of crown-of-thorns starfish have limited influence on the structural complexity of reef habitats in the short term compared to the immediate physical damage from storms, although the longer-term impacts may be similar [75]. For outbreaks of ciguatera to increase, there must be an increase in the amount of CTX that accumulates in fish that are eaten by people or a shift in the pattern of human consumption to higher-risk fish species.

**Figure 1:**
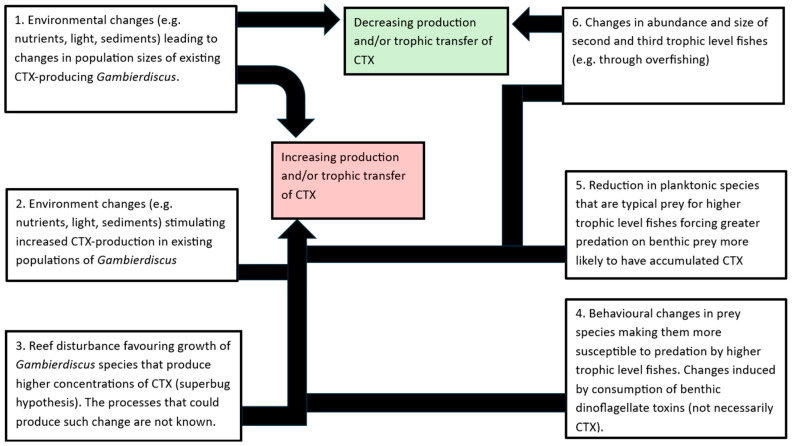
Six hypotheses for how reef disturbance causes or contributes to changes in the production and/or trophic transfer of CTX through marine food chains to affect the risk of ciguatera.

Changes that increase the risk of ciguatera could occur at each trophic level of marine food chains, or in the transfer processes between trophic levels, or combinations of these (Figure 1). At the base of the ciguatera food chain, these could occur through processes that:

1. Increase cell numbers (a bloom) of resident CTX-producing *Gambierdiscus*, and/or,
2. Increase cellular production of CTX in resident *Gambierdiscus* species or strains and/or,
3. Shift the dominance of the species/strains of resident *Gambierdiscus* from low CTX-producing to higher CTX-producing populations (super-bug hypothesis).

Additionally, changes within existing food chains that could increase the amount of CTX transferred between trophic levels could occur through processes that cause:

4. Behavioural changes in consumer species that ingest *Gambierdiscus* that increase their risk of predation and therefore increase the probability that CTX is transferred to higher trophic level fish more often consumed by people, and/or,

Ecosystem changes that act on food chains to:

5. Alter the diet of herbivorous or carnivorous fishes that increases the flux of CTX transferred to higher trophic levels, and/or,
6. Changes in abundance and/or size of reef fishes that alter the dynamics of reef food chains and the flux of CTX through them to human consumers. This could occur through natural processes such as variation in fish recruitment, and/or depletion of stocks from harvesting of marine resources.

In this paper, we review the evidence for these six potential mechanisms to increase ciguatera risk from disturbance to coral reefs and suggest a hypothesis where ecosystem changes (Section 2.5) could increase the flux of CTX to predatory fishes through a shortening of food chains that changes predation by groupers from prey species that predominately feed on planktonic organisms, to those that mostly feed on benthic organisms. However, the evidence for this hypothesis comes mostly from studies in the Pacific and Indian Oceans and may not apply to the Atlantic Ocean and adjacent waters. Our review assumes that *Gambierdiscus* and possibly *Fukuyoa* species are the principal origin of ciguatoxins and assumes other potential sources, such as cyanobacteria [76,77,78], contribute little to ciguatera risk [4].

## 2. Examination of the Six Potential Mechanisms to Increase Ciguatera Risk from Disturbance to Coral Reefs

### 2.1. Increase Cell Numbers (A Bloom) of Resident CTX-Producing Gambierdiscus

The proliferation of *Gambierdiscus* through cell growth is widely hypothesised to create an increased risk of ciguatera and is consistent with ciguatera appearing in the absence of any large-scale reef disturbance. There is a comprehensive literature on factors influencing the growth of *Gambierdiscus*, beginning with its culture and environmental response studies from the 1970s [79,80,81,82]. This research has only increased since the discovery in the 1990s that the genus consisted of more than one species [3,83]. To date, 19 species of *Gambierdiscus* have been recognized [3,84], with many of these species suggested capable of producing CTX analogs, although their cell concentrations vary considerably [3,16,17,85,86,87]. Therefore, early studies on *Gambierdiscus* growth and toxicity need to be interpreted cautiously as they assumed a monotypic genus (*G. toxicus*), with later studies showing differences across species [85]. For a *Gambierdiscus* bloom to increase the risk of ciguatera, the species proliferating must be a significant CTX producer, as growth of low-CTX producing species are unlikely to cause an increased risk of ciguatera [2,4,7,58,85,88,89,90]. The discovery of increasing numbers of *Gambierdiscus* species that produce different arrays and levels of CTX-analogs has parallels to research on *Alexandrium* spp. and the paralytic shellfish toxins they produce [91].

*Gambierdiscus* populations could bloom through an environmental change that increases cellular growth rates and/or through a reduction in herbivore grazing pressure [4,67]. As yet, there is no direct evidence of disturbance to coral reefs stimulating *Gambierdiscus* growth, although grazing pressure on algae can be reduced from increased sedimentation [92,93,94,95,96], which may be exacerbated by reef disturbance. Indirect evidence for an association between peak *Gambierdiscus* densities and disturbance was reported in French Polynesia, where *Gambierdiscus* population densities and bloom frequency increased after coral bleaching caused by elevated seawater temperatures [90]. Rongo and van Woesik [32] also found a correlation between reef disturbance and increased ciguatera risk in Rarotonga in the southern Cook Islands, but there was no associated analysis of *Gambierdiscus* populations. Disturbance of sediments and damage to reefs could release or resuspend bioavailable nutrients into the water column [97,98], stimulating *Gambierdiscus* growth, and this, along with the creation of new surfaces, have been suggestions for how reef disturbance could increase ciguatera risk. However, the impact of sedimentation on *Gambierdiscus* growth and toxin production has not been assessed. In the absence of physical disturbance, nutrients are often input from terrestrial runoff to inshore reefs, while on outer reefs, nutrient levels can be influenced by upwelling [99,100,101].

The creation of new surfaces on its own is unlikely to stimulate dinoflagellate growth but would provide increased area for growth of algal substrates (macroalgae, turf algae) for *Gambierdiscus*. However, these new surfaces would only lead to an increase in ciguatera risk if they were colonised by CTX-producing species that then proliferated and were then grazed by fish or invertebrates. Short-term increases in herbivore populations, including species often associated with causing ciguatera, such as the surgeonfish *Ctenochaetus striatus* and other acanthurids, can occur in response to increased filamentous algae on bleached corals, potentially through migration to feed on more abundant or preferred sources of food [102]. This short-term increase in localized herbivory could lead to increased accumulation of CTX into these herbivores, likely contributing to the patchy distribution of ciguatoxic fishes. In societies where herbivorous/detritivorous fishes are directly harvested for food, such as island nations in the Pacific Ocean, this could increase ciguatera risk [6]. Conversely, in areas such as the Great Barrier Reef, where herbivorous/detritivorous fishes are generally not harvested for food [103,104], any increased levels of CTX into the second trophic level may not always lead to an increased risk of ciguatera [4].

Cage experiments have shown that macroalgae on coral reefs can be limited by grazing [38], but there is little evidence that the spatial coverage of turf algae is similarly limited by grazing. Such limitation may be unlikely as turf algae can grow rapidly and colonise numerous substrates. New surfaces can be colonized by turf algae within weeks [48,49,105] and be dominant after six months [51]. While disturbance to coral reefs can create new surfaces for turf algae to proliferate that may favour increased grazing in the short term [102], any associated increase in sedimentation can change the structure and productivity of turfs such that herbivore grazing is suppressed [70]. In the Caribbean, overfishing of herbivores, along with eutrophication and the die-off of sea urchins and corals, has been associated with proliferation of macroalgae [106,107,108,109]. If the smaller population of herbivores created by overfishing reduced competition for algal substrates supporting *Gambierdiscus* populations, this could facilitate the funnelling of CTX into these now smaller populations of herbivores [4]. However, this mechanism is not necessarily associated with physical disturbance to the coral reef systems. The often-cited increase in macroalgae on disturbed coral reefs appears to be a feature of the Caribbean, with little evidence for a general increase on Indo-West Pacific reefs [52,110].

French Polynesia saw an increase in herbivores that controlled macroalgae that proliferated after reef disturbance [111]. This feedback response suggests increased grazing associated with increased food availability (macroalgae) [112]. Even though this was considered a rapid response, the changes were significant only over a time scale of years [111], which compounds the difficulty of trying to attribute causal relationships for ciguatera risk. In addition, the environmental outcomes from reef degradation resulting from interactions between corals, macroalgae, and fish (among other factors) are complex [43,113,114,115] that can vary depending upon herbivore/grazer biomass, herbivore size, and fish community structure [103,116,117], and possibly with the state (initial conditions) of the reef before the disturbance [118]. These complex interactions limit any analysis of the factors that are more likely to lead to increases in ciguatera risk, especially when underlying mechanisms may differ between regions.

### 2.2. Increase Cellular Production of CTX in Resident Gambierdiscus Species or Strains

The hypothesis that resident populations of *Gambierdiscus* can increase the production of CTX in response to an environmental stimulus is intuitively appealing as an explanation for the unpredictable nature of ciguatera outbreaks. The first experimental support for this hypothesis that specifically examined changes in presumed CTX concentrations in *Gambierdiscus* (cellular Na^+^-channel toxicity) was described by Sperr and Doucette [119], who reported enhanced ciguatoxicity under batch culture growth using differing N:P nutrient ratios. However, it is not clear how changes in nutrient ratios *per se* affect toxicity, especially if the cells are nutrient-replete, which may be likely in the exponential growth phase of batch culture, irrespective of which nutrient ratio the cells are growing under. Possibly, batch cultures with differing nutrient ratios may be more likely to affect toxin production as growth becomes limiting and cultures enter stationary growth phase. It is interesting that toxin production is often highest at low growth rates of *Gambierdiscus*, i.e., under conditions that limit cell division [119,120,121,122,123], including some strains grown under nitrogen-limiting conditions [120]. How these laboratory culture conditions relate to natural environmental changes that affect ciguatoxin production remains to be determined but may be inferred from the relative change in CTX concentrations in cultures of some strains of *Gambierdiscus*. The maximum relative change in CTX concentrations is ~2- to ~3-fold across many cultures [120,122,123,124]. Such variation needs to be factored into future models of the production and flow of CTX in food chains (see recent modelling by Holmes and Lewis [5,6] and Parsons et al. [7]). It is possible that a 2–3-fold change in cellular CTX concentration induced by environmental factors alone could influence ciguatera risk, either up or down, depending on the specific suite of environmental factors at play.

Nutrients can be released into the water column from the resuspension of sediments [97,98]. The type of disturbance impacting a reef will affect the quantity and duration of nutrient release, from the chronic, long-term effects of land-based agriculture to short-term pulses such as from storms or ongoing dredging. Episodic disturbance could lead to nutrient pulses that promote *Gambierdiscus* blooms that then increase in toxicity as nutrients become limiting [120] as ambient nutrient concentrations return to the low levels typical of coral reef waters [101]. This would correspond to a two-step process to increase CTX load on a reef.

Changes in other environmental parameters, such as salinity and temperature, have also been shown to vary CTX concentrations in *Gambierdiscus* [121,122], but physical disturbances to coral reefs are unlikely to significantly alter these parameters in the short term. Vacarizas et al. [122] did find the highest CTX production for *G. carpenteri* growing under low light conditions. The light (photon flux density) transmitted through the water column would likely be reduced by increased turbidity associated with reef disturbance, although this will depend in part on the type and duration of the disturbance. While reduction in light may affect CTX production of an existing bloom of *Gambierdiscus*, it may also limit nutrient-driven growth. The decrease in light would presumably need to be of considerable magnitude, as the light received by benthic organisms typically changes constantly throughout the day and varies with season, water depth, cloud cover, and turbidity, all of which affect the clarity of the water column, and *Gambierdiscus* can orientate itself within the light environment [125]. Most culture studies do not attempt to mimic this variability of the light environment in the wild, relying instead on continuous fluorescent or LED lighting with unchanging day-night photoperiods. It would be interesting to test whether changes in light alter the toxicity of cultures instead of the typical practice of acclimating them to new light regimes before analysing for toxicity. Any effect from a reduced light regime would only be relevant over limited time scales for the transfer of CTX to herbivorous fishes, given the inverse relationship between increasing turbidity and herbivorous fish abundance on coral reefs, likely because of the impact of reduced light on the biomass and nutritional value of turf algae to herbivorous fishes [126]. Ciguateric predatory fishes have been caught from deep water (>200 m) where light levels are low; however, the origin of the CTX was suggested to be *Gambierdiscus* from shallower depths [127]. As suggested by Chinain et al. [85], further research is required to understand how changes in environmental conditions can affect toxin production across different species and strains of *Gambierdiscus*.

The possibility of biotic factors influencing CTX production and structural modifications of toxins also needs to be considered. Wang et al. [128] reported that co-occurring species of bacteria could increase or decrease CTX production in *Gambierdiscus*. There is also evidence that *Gambierdiscus* can acquire nutrients through mixotrophy [129,130]. However, studies on whether heterotrophic nutrition and its relative contribution to homeostasis can affect production of CTX by *Gambierdiscus* are lacking. There is also an increasing literature on how certain grazers can induce toxin production in populations of planktonic diatoms and dinoflagellates [131,132,133,134,135,136,137]. However, we are not aware of any such studies on CTX production in *Gambierdiscus* or other benthic dinoflagellates. If grazing by fish or invertebrates could induce increased production or accumulation of CTX in adjacent (down current) populations of *Gambierdiscus*, it could increase the CTX load on a reef. Inducible defence mechanisms to deter grazing are common in plants [138]. However, any such response for benthic dinoflagellates would need to be rapidly inducible to provide time to act as a grazing deterrent. Additionally, any such inducible deterrent to herbivore foraging could also operate for the algal substrate as for its epiphytic dinoflagellates. However, it is not clear how such biotic mechanisms would relate to reef disturbance.

In an early attempt during the 1980s to examine if reef disturbance could directly stimulate CTX production by *Gambierdiscus* through a hypothesised CTX-inducing factor [88], we added seawater extracts of the ground-up tips of living staghorn coral (*Acropora* sp.) collected from Flinders Reef in south-east Queensland [139] to cultures of *Gambierdiscus* isolated from Flinders Reef and the Great Barrier Reef [89]. These were cultures from which we could not initially detect any CTX by mouse bioassay [89]. After one week, we harvested the spiked cultures but again could not detect any CTX by mouse bioassay (unpublished results), so we could not find any support for the hypothesised CTX-inducing factor. These experiments were carried out at a time when *Gambierdiscus* was assumed to consist of only one species (*G. toxicus*), so we cannot attribute the species used in our experiments. In summary, while individual factors arising from damage or disturbance to coral reefs could change environmental conditions to affect the growth of *Gambierdiscus*, there is insufficient evidence of any consistent processes enhancing the production of CTX in resident populations of *Gambierdiscus* species or strains.

### 2.3. Shift the Dominance of the Species/Strains of Resident Gambierdiscus from Low CTX-Producing to Higher CTX-Producing Populations (Super-Bug Hypothesis)

Many species of *Gambierdiscus* are thought to produce CTX, but the cell concentrations can vary many-fold [3,16,17,85,86,87,124]. The existence of what we called super-producing strains of *Gambierdiscus* [2,89,140,141] originated from the detection of large differences in CTX concentrations between cultured and wild *Gambierdiscus* with large differences subsequently reported between and within species [3,86,123,124,142]. This concept of super-producing species/strains has since been conceptualized as the “superbug” hypothesis [7,143]. The highest-known producers of CTX analogs (toxin cell quotas) are *G. polynesiensis* in the Pacific Ocean [87,123,124,144] and *G. excentricus* and *G. silvae* in the Atlantic [121,145,146,147,148,149]. These three species can have cell toxin quotas >1,000-fold higher than other species examined, including *G. caribaeus* and *G. carolinianus* [86,121,123,147]. This inter-species range of CTX concentrations is far greater than the ~2–3-fold range known from intra-species studies of CTX concentrations quantified across different growth phases of cultures [120,122,123,124].

*Gambierdiscus* populations have long been known to vary seasonally on coral reefs [7,66,86,90,138,150,151], although only recently has it been suggested that ciguatoxicity can also vary seasonally in an inverse relationship with cell abundance [151], possibly indicating greater toxicity under growth limiting conditions. The CTX-load of *Gambierdiscus* populations do not necessarily correspond to cell numbers [2,4,86,88,89,90,151,152], with CTX-production initially suggested to vary between strains [2,88,89,90] and later shown to vary between and within species [3,16,17,85,87,121,124,142,146]. Sites can host multiple *Gambierdiscus* species [86,143], with the species mix changing through time [143], so it is not unexpected that ciguatera risk, as it relates to CTX-production, can also change through time. It has long been known that different *Gambierdiscus* cultures isolated from the same site and at the same time can have very different CTX toxicities [89]. However, there is currently no evidence for disturbance to coral reefs favouring the proliferation of “superbug” species, which shift the dominance of low CTX-producers to higher CTX-producing species of *Gambierdiscus*.

### 2.4. Behavioural Changes in Consumer Species That Ingest Gambierdiscus That Increase Their Risk of Predation and Therefore Increase the Probability That CTX Is Transferred to Higher Trophic Level Fish More Often Consumed by People

Many groupers are opportunistic carnivores that prey on a variety of fish and invertebrate species [153,154,155], so if herbivores (fish or invertebrates) were intoxicated by CTX and/or other toxic-metabolites produced by benthic dinoflagellates when feeding on high populations of *Gambierdiscus*, this could provide a mechanism for facilitating predation on CTX-contaminated prey [2,4]. Two Caribbean surgeonfishes (*Acanthurus bahianus* and *A. chirurgus*) fed a gel diet containing a *Gambierdiscus* species became disorientated and lost equilibrium [156], behaviour that in the wild would greatly increase their chance of predation. However, Magnelia et al. [156] found that surgeonfish could acclimate to feeding on a fixed dose of *Gambierdiscus,* suggesting that the intoxicating effects and risk of opportunistic predation from carnivorous fish species may be greatest after they feed on a sudden population increase (bloom) of *Gambierdiscus*. This may be a mechanism by which occasional blooms of ciguatoxin-producing benthic dinoflagellates lead to the production of ciguateric predatory reef fish species (reviewed by Holmes et al. [4]).

Clausing et al. [8,157] found that juvenile spotted unicornfish (*Naso brevirostris*) did not display any signs of intoxication when fed a gel diet containing *G. polynesiensis*. This surgeonfish accumulates CTX in the wild at Nuka Hiva (Marquesas archipelago, French Polynesia), although locals have considered it an edible species [158] with juveniles and sub-adults feeding on benthic macroalgae whereas adults tend to feed on gelatinous zooplankton [8,159,160]. However, it is likely that the behavioural response of surgeonfishes to feeding on substrates supporting *Gambierdiscus* populations depends upon the species and size of the herbivore consuming them, the cellular concentration of CTX (or other metabolite(s) inducing signs of intoxication in the herbivore), and the number of cells being consumed [4,6]. The absence of abnormal swimming behaviour in dusky groupers (*Epinephelus marginatus*) fed naturally CTX-contaminated fish flesh [161] suggests that the induction of abnormal behaviour is species-specific or the result of fish (especially herbivores) consuming toxic metabolites from *Gambierdiscus* other than CTX. Modelling by us [6] suggests that in some circumstances, more *Gambierdiscus* cells may be consumed daily by surgeonfishes that feed on turf algae than in the experimental protocol by Clausing et al. [8,157]. However, the ability of models to predict CTX transfer through trophic levels is constrained by a lack of experimental data [4,5,6,7,8], with more experiments like those of Clausing et al. [8,157] needed to produce better models. Currently, behavioural changes induced by grazers/herbivores feeding on toxic metabolites produced by benthic dinoflagellate cannot be directly linked to disturbance to coral reefs.

### 2.5. Change in the Diet of Herbivorous or Carnivorous Fishes That Increases the Flux of CTX Transferred to Higher Trophic Levels

Groupers (such as species of *Plectropomus*, and *Epinephelus*) are carnivorous Serranid fishes targeted by fishers for food that also often cause ciguatera poisoning [3,4]. On Queensland’s Great Barrier Reef, the common coral trout (*P. leopardus*) is the focus of the commercial fin-fish fishery [162] and is also one the principal causes of ciguatera in Queensland, Australia [1,6,163]. While groupers tend to be opportunistic predators, different species typically prey on benthic and planktonic species to varying degrees. For example, *P. leopardus* predominantly prey on plankton-feeding fish on the Great Barrier Reef [154,155,164,165] as well as other mesopredators [166]. In contrast, barcheek coral trout (*P. maculatus*), which tend to be found on more inshore reefs than *P. leopardus*, often consume a greater proportion of benthic prey [155,167], although there is considerable overlap in the distributions of these two species [168], and they can hybridize [169]. Plankton-based diets are unlikely to be a major source for the transfer of CTX to carnivores, although the rapid colonisation of benthic screen assays [54,55] indicates that at least some *Gambierdiscus* are tycoplanktonic, so they possibly could be consumed by zooplankton. Zooplankton are thought to be the principal diet of flying fishes [170], and the recent finding of CTX in glider flying fish (*Cheilopogon atrisignis*) [171] possibly suggests that tycoplanktonic *Gambierdiscus* could be a secondary food chain for the transfer of CTX. As the major CTX found in the flying fish was the less-oxidised P-CTX-2, it supports the contention that the toxin was consumed from an organism close to the base of the ciguateric food chain [4]. Intuitively, grouper species that principally prey on plankton-feeding fishes should be less likely to accumulate CTX. However, behavioural changes in potential prey species (reviewed in Section 2.4) could be a mechanism that increases the flow of CTX into opportunistic carnivores, including *P. leopardus* [4,6]. Recent studies in the Maldives (Indian Ocean) on groupers (Serranids) and tropical snappers (Lutjanids) from coral reefs support their reliance on planktonic prey [172].

Reefs around the Keppel Island group on the southern Great Barrier Reef were typified by exceptionally high coral cover, but much of it was lost due to coral bleaching and increased inundation by sediment-laden, freshwater flood plumes [173]. Long-term monitoring of these reefs demonstrated that, as coral cover declined, there was a decrease in the biomass of prey for mesopredators such as groupers, and a shift in dominant prey species from pelagic, plankton-feeding damselfishes to territorial benthic, algal-feeding damselfishes [173]. This resulted in an effective shortening of the food chain for *P. maculatus* from longer planktonic dominant food chains to shorter benthic dominant food chains [173]. Such a switch would suggest that these predators would be feeding on prey that had a greater likelihood to have ingested epiphytic dinoflagellates such as *Gambierdiscus* and *Fukuyoa* and, therefore, more likely to accumulate CTX if it was being produced by the local benthic dinoflagellates. A similar shortening of food chains, with a switch to greater feeding on benthic-feeding prey, was also reported for the grouper *Cephalopholis argus* after disturbance to reefs in the Seychelles, Indian Ocean [174]. These findings suggest that this may be a mechanism that can increase the risk for CTX to enter the food chain of mesopredatory fishes that are the focus of many reef fisheries. Several other studies in the Pacific and Indian Oceans have also reported reductions in plankton-feeding fishes after disturbance to coral reefs [175,176,177], indicating that predators could be forced to seek alternate, benthic sources of nutrition. This shift in predation could lead to an increased flux of CTX to higher trophic levels without any increase in the production of CTX or in the toxicity of herbivores.

The shift in dominant prey species for groupers from planktonic-feeding damselfishes to benthic-feeding species [173] suggests an avenue for future research for the food chain transfer of CTX. To date, much ciguatera research has focussed on surgeonfishes as the vectors for the transfer of CTX from benthic dinoflagellate source to carnivorous fish such as groupers (reviewed by Holmes et al. [4]). However, early reports from the Pacific included damselfishes among ciguateric species [21,22,178,179]. Additionally, the omnivorous damselfish *Stegastes diencaeus* eats benthic microalgae and crustaceans [180] and has tested positive for CTX in the Caribbean [181]. It has also been suggested that the loss of larger-bodied herbivorous fishes can lead to increased biomass and abundance of algal-farming damselfishes on reefs [108], which could possibly increase the flux of CTX into food chains.

Morillo-Velarde et al. [61] reported changes in trophic pathways for predatory fishes on degraded reefs in the Caribbean, but unlike the studies of Hempson et al. [173,174], there was no shortening of food chain length. Also, in contrast to the studies on degraded reefs in the Pacific and Indian Oceans, on degraded Caribbean reefs, the major carbon source for the carnivorous fish species studied changed from benthic sources (especially turf algae and epiphytes) to particulate organic matter that would likely include a greater planktonic component. In this case, it would suggest that disturbance to coral reefs in the Caribbean may not lead to an increased risk of CTX being transferred along food chains into groupers, as hypothesised for the Pacific and Indian Oceans. The limited studies with contrasting conclusions from the Caribbean Sea compared to the Pacific and Indian Oceans indicate the need for more research and raises the possibility that there may be different processes controlling the flow of CTX along food chains in different geographic regions. However, it would not be surprising if there were differences between the Pacific and Caribbean pathways for CTX accumulation into higher trophic level fishes on coral reefs, as their benthic ecosystems are reported to function in fundamentally different ways [52,64,182]. This has arisen in part because of their different biogeographical histories, which has led to a more than 3-fold difference in the diversity of fishes between the Indo-West Pacific and Caribbean regions [52,182,183] and in how different fish assemblages use reef and adjacent habitats [184]. This affects the ecosystem functioning of herbivory, with the diversity of herbivorous fishes of the Western Atlantic considered depauperate compared with the Indo-West Pacific [52,185,186]. Thus, we expect that the mechanisms controlling the development of ciguateric fishes will vary between regions.

The impacts arising from the biogeographical separation of tropical waters between the major oceans on how ciguatera and ciguatoxicity develops across regions is a matter of debate. Experimental evidence and recent modelling suggest that only high-CTX-producing *Gambierdiscus* species pose a major risk for producing ciguateric fishes capable of poisoning people, especially for P-CTX-1 analogs [2,4,5,6,7,8,58,85,88,89,90,157]. Currently, high-CTX-producing species of *Gambierdiscus* are only known from the Pacific and Atlantic Oceans but can be reasonably inferred to also occur in the Indian Ocean. The tropical waters of the Pacific and Atlantic Oceans have been geographically separated for ~3 million years by the Isthmus of Panama [187]. In contrast, the flow of tropical water between the Pacific and Indian Oceans may not have been as constrained by land barriers as sea levels changed during ice ages. Even during the last glacial maximum (~20,000 years ago), tropical waters of these two oceans were likely still connected via straits between the Sunda Shelf (encompassing much of Southeast Asia) and Sahul (Australia, Papua New Guinea) [188,189]. Thus, a connection potentially allowing the transfer of *Gambierdiscus* on floating debris [190] or by tycoplanktonic cells transported incrementally on tidal currents via a stepping-stone process [191] may have been possible between these two oceans for considerably longer than between the tropical Pacific and Caribbean. However, many of the same *Gambierdiscus* species are known from both the Pacific and Atlantic Oceans [3], suggesting limited endemicity among this group. It is interesting to speculate on the extent to which biogeographical differences have driven the biochemistry of CTX analogs between and across different oceans. Loeffler et al. [192] have suggested the presence of P-CTX3C in fishes from the Indian Ocean (previously, this analog has been only known from the Pacific Ocean), although their result requires confirmation given the uncertainty in tracing the origin of contaminated fish frozen and stored for ~3 years after capture and processing. Similarly, putative analogs of I-CTX have been recently detected from groupers caught from Okinawa that were also contaminated with P-CTX-1 [193]. Analysing the population genetics of fish to confirm the region of capture as well as the CTX-analogs produced by *Gambierdiscus* isolated from reefs in waters that have had long-term connections between the tropical Pacific and Indian oceans will help confirm that the distribution of P- and I-CTX analogs are indeed not restricted to one Ocean. Based upon maps of changing shorelines over the Pleistocene epoch [189], this could include reefs in and around the Banda and Savu Seas, adjacent to Indonesia and Timor Leste. This area is part of the Indonesian Throughflow, a series of currents that form the only tropical pathway connecting the global oceans and that have a crucial role in heat and water exchange between the Pacific and Indian Oceans [194,195]. As this drives water through Indonesia into the Indian Ocean it could provide a transport pathway for *Gambierdiscus* from the Pacific into the northeastern Indian Ocean. We are unaware of any studies of *Gambierdiscus* from this region; see recent review of ciguatera in the Indian Ocean [196]. However, benthic reef fish contaminated with P-CTX-1 are known to occur in the Arafura Sea [197], adjacent to the Indonesian Throughflow. We therefore predict that P-CTX-1-producing dinoflagellates likely occur in the northeastern Indian Ocean.

### 2.6. Changes in Abundance and/or Size of Reef Fishes That Alter the Dynamics of Reef Food Chains and the Flux of CTX Through Them to Human Consumers: This Could Occur Through Natural Processes Such as Variation in Fish Recruitment and/or Depletion of Stocks from Harvesting of Marine Resources

The conceptual model for the origin of ciguatera is based on a simplistic, linear model where *Gambierdiscus* blooms are incidentally consumed by herbivorous fish, which are preyed on by carnivorous fish, that are caught and eaten by humans, who are then poisoned. This is because the analysis of the role that ecological processes play in the production of CTXs and their transfer/bioconversion across trophic levels to cause ciguatera is still in its infancy and mostly relies upon data from ecological studies not directly related to ciguatera. We only use discrete trophic levels to describe the transfer of CTX along food chains in this review as our focus is on the transfer of toxins from source (*Gambierdiscus*) through intermediate vectors (invertebrate and/or herbivore), to predators. The estimation of trophic levels using stable isotopes integrates the assimilation of energy or mass flow through all the different trophic pathways leading to the organism [198], which produces non-integer trophic level numbers that have been recently used to estimate trophic magnification factors for CTX in fish [171]. However, this may not be representative of toxin transfer dynamics as the integrated sources of energy ingested can overwhelm those corresponding to the subset associated with toxin transfer, with δ^15^N and δ^13^C potentially reflecting the major nutritional sources rather than those associated with the production and transfer of CTX. For example, an initial vector in the CTX transfer process, such as the surgeonfish *Ctenochaetus striatus,* can have an equal or higher trophic level number than some groupers (*Epinephelus maculatus*, *Variola albimarginata*), their potential predators [171]. *Ctenochaetus striatus* is a detritivore that feeds by using its mouthparts to brush detritus aggregates from turf algae [95,199,200] and, in the process, ingests *Gambierdiscus* (reviewed by Holmes et al. [4]). The higher trophic number for *C. striatus* and some other herbivores [171] may occur because the substrate could include small invertebrates and material decomposing (recycled) from higher trophic levels, including fish faeces [199,201]. It is possible that the aggregated detrital material could also contain CTX, but this would be a different food chain pathway from the direct assimilation of CTX from *Gambierdiscus*.

The abundance and size distribution of grazers and predators in the ciguateric food chain are likely key factors affecting the transfer of CTX along food chains that lead to ciguateric fishes. Fish abundance can change considerably through natural processes such as variability in annual fish recruitment and predation, as well as from the human pressures of habitat modification, climate change, and fishing. The life stage at which fish accumulate CTX, as well as the frequency of any subsequent accumulations, are also likely important factors influencing whether sufficient toxin is transferred through trophic levels to produce a fish that can poison people. Additionally, dilution of toxicity through fish growth and depuration can reduce toxin concentrations in fish, although modelling suggests this may not occur quickly enough to reduce the risk of ciguatera from adult fish (reviewed by Holmes et al. [4]) with evidence of very rapid rates of depuration in juvenile fish [202,203].

In the Caribbean, long-term disturbance to coral reefs has resulted partly from the die-off of corals and sea urchins from disease and the effects of overfishing [106]. Parrotfishes (Labridae, Scarinae) are thought to contribute to the control of macroalgae, although some studies suggest that many are microphages [204,205] that may be more controlled by the benthos through bottom-up processes rather than exerting top-down control of macroalgae on coral reefs [206]. Parrotfish are targeted for food throughout much of the Caribbean, in part because of their greater availability relative to depleted stocks of higher-value large predatory fishes such as groupers and tropical snappers [207]. It is the larger-sized fish that contribute disproportionately to the control of macroalgae in the Caribbean if modelling by Bozec et al. [208] is correct in concluding that even modest (>10%) harvesting of large (>30 cm) parrotfish can reduce the recovery and resilience of Caribbean reefs. If the combination of overfishing and increased macroalgal biomass increases the risk of ciguatera, it would be reasonable to expect an increase in the rate of ciguatera poisoning to have occurred after the general increase in macroalgae in the Caribbean (assuming the relative impact of all other factors remained unchanged). This would not necessarily correspond to a uniform rate of increase, as the incidence of ciguatera varies greatly across the region [209]. However, we are not aware of any studies reporting significant increases in ciguatera occurring after the increase in macroalgae in the Caribbean, although there are suggestions of increases associated with warming sea surface temperatures [210] and increased storm frequency [33].

In the Pacific, grazing pressure increases non-linearly with increasing grazer biomass [211], although grazing pressure for a given biomass tends to be greatest with smaller fish sizes [212]. So, targeted fishing that removes larger-sized herbivorous fish may increase overall grazing pressure if the relaxation of competition from larger-sized herbivores leads to a relative increase in the cohort of smaller-sized herbivores. Although grazing pressure appears to be greatest when the population structure of larger-sized grazers is truncated [212], the ecological processes of herbivory vary significantly across the Pacific [211]. It has even been suggested that a population structure with smaller-sized parrotfish could counterintuitively favour macroalgal growth through the greater removal of epibionts from the surface of macroalgae [213].

In French Polynesia, high fishery value combined with slow life histories predispose unicornfishes (Acanthuridae, surgeonfishes) to overexploitation compared with parrotfishes [116]. If overfishing of high-value unicornfishes led to an increase in macroalgae then the increased substrate could allow for larger populations of *Gambierdiscus* to be consumed by the smaller remaining population of unicornfishes [4,6]. This provides a mechanism for the development of ciguatera, especially if compensatory processes do not operate among surgeonfishes and parrotfishes in the Pacific [211]. That is, the reduction in rates of herbivory from the loss of one type of herbivore is not offset by increases from another. Stocks of surgeonfishes (including unicornfishes) on the Great Barrier Reef are not directly affected by fishing as they have little commercial value for human consumption [103,104], and aggregate pressure from subsistence fishing on the Great Barrier Reef has been minimal. This is likely a contributing factor to the lower incidence of ciguatera from Australia compared to French Polynesia. The minimal commercial harvesting (and discouragement of recreational harvesting) of parrotfishes on the Great Barrier Reef may be an additional factor that reduces the risk of ciguatera on the east Australian coast relative to the Caribbean and Pacific Island nations.

While overfishing is usually related to human population pressures along with access to technologies that increase efficiency, the outcomes for any ecosystem are also dependant on cultural norms for consumption of seafood (that also changes over time). In many parts of the Pacific, overfishing of all trophic levels has occurred, with depletion of large-bodied predators taking place along with, or followed by, removal of large-bodied herbivores, especially macroalgal browsers [214]. This can lead to a growing dominance of small-bodied herbivores on coral reefs [215,216,217]. Interestingly, the removal of large browsers also favours increased biomass and abundance of algal farming damselfish [108], which could be a mechanism for the transfer of CTX to the remaining higher trophic level predators [4]. While overfishing has a long-term impact on coral reefs, it is not clear if disturbances to coral reefs can interact with extractive fishing to exacerbate ciguatera. The role of small herbivores as potential vectors for CTX transfer requires further investigation.

## 3. Conclusions

The provision of new surfaces after damage to coral reefs remains a viable hypothesis for increased ciguatera risk if the algae subsequently colonising the damaged reef are, in turn, colonised by a *Gambierdiscus* species that produce significant levels of CTX. Given how quickly benthic screens are colonised by *Gambierdiscus* [54,55], it seems likely that the new algal substrates will almost certainly be rapidly colonised by *Gambierdiscus* species. However, the requirement that these colonising *Gambierdiscus* species include at least one significant CTX-producer may limit which *Gambierdiscus* blooms increase ciguatera risk on reefs. In what appears common to both the Pacific and Atlantic Oceans, multiple *Gambierdiscus* species often co-occur on reefs, but the population densities of high toxin producers are frequently low [86,143]. The next step in the food chain to produce a ciguatoxic fish requires the proliferation of high-CTX-producing species or strains of *Gambierdiscus* growing on the new substrate. While the ambient environmental conditions likely control the capacity for *Gambierdiscus* to bloom, the CTX load on a reef may be influenced by factors that limit growth but increase cellular concentrations of CTX, with grazing pressure potentially limiting the capacity for *Gambierdiscus* populations to bloom [4,6].

For an increased risk of ciguatera, the substrate supporting a bloom of high CTX-producing *Gambierdiscus* should be preferentially consumed and CTX transferred to subsequent trophic levels. Indeed, there is evidence that new algal substrates on damaged reefs can be preferentially grazed [102,113]. In societies that consume herbivores, grazers and/or detritivores, this could increase the risk of ciguatera, although such risk would be partially offset over time by fish growth (toxin-dilution) and depuration [4]. Where carnivorous fish species are the main cause of ciguatera, CTX must be transferred from herbivores/grazers/detritivores to carnivores, with the number and toxicity of second trophic level fish greatly affecting CTX levels in the carnivorous fishes that ultimately poison people [5,6]. There is also evidence that disturbance to coral reefs in the Pacific and Indian Oceans can reduce the availability of prey species that predominantly feed on planktonic food sources, resulting in a shift to benthic-feeding prey for groupers [173,174], which are more likely to be contaminated with CTX on reefs supporting CTX-producing populations of *Gambierdiscus*.

Despite progress, more research is needed to understand the ecological processes that generate ciguateric fishes on both undisturbed and disturbed reefs. Could the rapid recovery of coral cover (but not necessarily function) that often occurs after damage to reefs [218,219] play a role in limiting ciguatera outbreaks? The proportion of toxic fish at Muroroa Atoll (French Polynesia) remains high (87%) despite nearly 30 years of recovery from the damage caused by nuclear testing [86]. It seems obvious that such tests would have caused significant damage to the surrounding reef, but although the last nuclear test at Muroroa Atoll was in late 1995, the *Gambierdiscus* species so far found there have limited ability to produce CTX (*G. australes* and *G. pacificus*) [86]. The difficulty in identifying the source of the benthic CTX load supporting the high proportion of toxic fish at Muroroa atoll or the ecological drivers maintaining toxicity highlights the challenges of understanding the drivers of increased ciguatera risk. We propose that key ecological processes controlling the production of ciguateric fishes remain to be elucidated, with our review suggesting that these likely vary at a regional level. As the world undergoes its fourth global coral bleaching event on record [220], it is important to better understand the key drivers affecting ciguatera risk that operate at these regional levels to mitigate the impact of ciguatera on low-income nations that rely so heavily on coral reefs for revenue and nutrition.

## Data Availability

No new data were created or analyzed in this study.

## References

[1] Gillespie N.C., Lewis R.J., Pearn J.H., Bourke A.T.C., Holmes M.J., Bourke J.B., Shields W.J. (1986). Ciguatera in Australia: Occurrence, clinical features, pathophysiology and management. Med. J. Aust..

[2] Lewis R.J., Holmes M.J. (1993). Origin and transfer of toxins involved in ciguatera. Comp. Biochem. Physiol..

[3] FAO, WHO (2020). Report of the Expert Meeting on Ciguatera Poisoning: Rome, 19–23 November 2018.

[4] Holmes M.J., Venables B., Lewis R.J. (2021). Critical review and conceptual and quantitative models for the transfer and depuration of ciguatoxins in fishes. Toxins.

[5] Holmes M.J., Lewis R.J. (2022). Origin of ciguateric fish: Quantitative modelling of the flow of ciguatoxin through a marine food chain. Toxins.

[6] Holmes M.J., Lewis R.J. (2023). Model of the origin of a ciguatoxic grouper (*Plectropomus leopardus*). Toxins.

[7] Parsons M.L., Richlen M.L., Smith T.B., Anderson D.M., Abram A.L., Erdner D.L., Robertson A. (2024). CiguaMOD I: A conceptual model of ciguatoxin loading in the Greater Caribbean Region. Harmful Algae.

[8] Clausing R.J., Gharbia H.B., Sdiri K., Sibat M., Rañada-Mestizo M.L., Lavenu L., Hess P., Chinain M., Bottein M.-Y.D. (2024). Tissue distribution and metabolization of ciguatoxins in an herbivorous fish following experimental dietary exposure to *Gambierdiscus polynesiensis*. Mar. Drugs.

[9] Murata M., Legrand A.-M., Ishibashi Y., Fukui M., Yasumoto T. (1990). Structures and Configurations of Ciguatoxin from the Moray Eel *Gymnothorax javanicus* and Its Likely Precursor from the Dinoflagellate *Gambierdiscus toxicus*. J. Am. Chem. Soc..

[10] Satake M., Murata M., Yasumoto T. (1993). The structure of CTX3C, a ciguatoxin congener isolated from *Gambierdiscus toxicus*. Tetrahedron Lett..

[11] Oshiro N., Nagasawa H., Watanabe M., Nishimura M., Kuniyoshi K., Kobayashi K., Sugita-Konishi Y., Asakura H., Tachihara K., Yasumoto T. (2022). An Extensive Survey of Ciguatoxins on Grouper *Variola louti* from the Ryukyu Islands, Japan, Using Liquid Chromatography–Tandem Mass Spectrometry (LC-MS/MS). J. Mar. Sci. Eng..

[12] Oshiro N., Nagasawa H., Nishimura M., Kuniyoshi K., Kobayashi N., Sugita-Konishi Y., Ikehara T., Tachihara K., Yasumoto T. (2023). Analytical Studies on Ciguateric Fish in Okinawa, Japan (II): The Grouper *Variola albimarginata*. J. Mar. Sci. Eng..

[13] Lehane L., Lewis R.J. (2000). Ciguatera: Recent advances but the risk remains. Int. J. Food Microbiol..

[14] Lewis R.J., Vernoux J.-P., Brereton I.M. (1998). Structure of Caribbean ciguatoxin isolated from *Caranx latus*. J. Am. Chem. Soc..

[15] Hamilton B., Hurbungs M., Vernoux J.P., Jones A., Lewis R.J. (2002). Isolation and characterisation of Indian Ocean ciguatoxin. Toxicon.

[16] Mudge E.M., Miles C.O., Ivanova L., Uhlig S., James K.S., Erdner D.L., Fæste C.K., McCarron P., Robertson A. (2023). Algal ciguatoxin identified as source of ciguatera poisoning in the Caribbean. Chemosphere.

[17] Mudge E.M., Robertson A., Uhlig S., McCarron P., Miles C.O. (2023). 3-epimers of 1 Caribbean ciguatoxins in fish and algae. Toxicon.

[18] Pottier I., Lewis R.J., Vernoux J.-P. (2023). Ciguatera Fish Poisoning in the Caribbean Sea and Atlantic Ocean: Reconciling the Multiplicity of Ciguatoxins and Analytical Chemistry Approach for Public Health Safety. Toxins.

[19] Estevez P., Oses-Prieto J., Castro D., Penin A., Burlingame A., Gago-Martinez A. (2024). First detection of algal Caribbean ciguatoxin in Amberjack causing ciguatera poisoning in the Canary Islands (Spain). Toxins.

[20] Miles C.O., Burton I.W., Lewis N.I., Robertson A., Giddings S.D., McCarron P., Mudge E.M. (2024). Isolation of Caribbean Ciguatoxin-5 (C-CTX5) and confirmation of its structure by NMR spectroscopy. Tetrahedron.

[21] Halstead B.W., Bunker N.C. (1954). A survey of the poisonous fishes of Johnston Island. Zoologica.

[22] Halstead B.W., Schall D.W. (1958). A report of the poisonous fishes of the Line Islands. Acta Trop..

[23] Randall J.E. (1958). A review of ciguatera, tropical fish poisoning, with a tentative explanation of its cause. Bull. Mar. Sci..

[24] Cooper M.J. (1964). Ciguatera and other marine poisoning in the Gilbert Islands. Pac. Sci..

[25] Helfrich P., Piyakarnchana T., Mikes P.S. (1968). Ciguatera fish poisoning. I. The ecology of ciguateric reef fishes in the Line Islands. Occas. Pap. Bernice P Bish. Mus..

[26] Bagnis R. (1969). Naissance et développment d’une flambée de ciguatera dans un atoll des Tuamotu. Rev. Corps Santé.

[27] Bagnis R. (1994). Natural versus anthropogenic disturbances to coral reefs: Comparison in epidemiological patterns of ciguatera. Mem. Qld. Mus..

[28] Banner A.H., Jones A.O., Endean R. (1976). Ciguatera: A Disease from Coral Reef Fish. Biology and Geology of Coral Reefs.

[29] Bagnis R., Bennett J., Barsinas M., Drollet J.H., Jacquet G., Legrand A.M., Cruchet P.H., Pascal H. Correlation between Ciguateric Fish and Damage to Reefs in the Gambier Islands (French Polynesia). Proceedings of the 6th International Coral Reef Symposium Executive Committee.

[30] Ruff T. (1989). Ciguatera in the Pacific: A link with military activities. Lancet.

[31] Rongo T., van Woesik R. (2011). Ciguatera poisoning in Rarotonga, southern Cook Islands. Harmful Algae.

[32] Rongo T., van Woesik R. (2013). The effects of natural disturbances, reef state, and herbivorous fish densities on ciguatera poisoning in Rarotonga, southern Cook Islands. Toxicon.

[33] Gingold D.B., Strickland M.J., Hess J.J. (2014). Ciguatera Fish Poisoning and Climate Change: Analysis of National Poison Center Data in the United States, 2001–2011. Environ. Health Perspect..

[34] Rarai A., Webber E., Ruben J., Parsons M. (2024). Indigenous knowledge with science forms an early warning system for ciguatera fish poisoning outbreak in Vanuatu. Commun. Earth Environ..

[35] Lewis N.D. (1986). Epidemiology and impact of ciguatera in the Pacific—A review. Mar. Fish. Rev..

[36] Banner A.H., Humm H.J., Lane C.E. (1974). The biological origin and transmission of ciguatoxin. Bioactive Compounds from the Sea.

[37] Belliveau S.A., Paul V.J. (2002). Effects of herbivory and nutrients on the early colonization of crustose coralline and fleshy algae. Mar. Ecol. Prog. Ser..

[38] Hughes T.P., Rodrigues M.J., Bellwood D.R., Ceccarelli D., Hoegh-Guldberg O., McCook L., Moltschaniwsky N., Pratchett M.S., Steneck R.S., Willis B. (2007). Phase shifts, herbivory, and the resilience of coral reefs to climate change. Curr. Biol..

[39] Mellin C., McNeil M.A., Cheal A.J., Emslie M.J., Caley M.J. (2016). Marine protected areas increase resilience among coral reef communities. Ecol. Lett..

[40] Stuart-Smith R.D., Brown C.J., Ceccarelli D.M., Edgar G.J. (2018). Ecosystem restructuring along the Great Barrier Reef following mass coral bleaching. Nature.

[41] Bellwood D.R., Pratchett M.S., Morrison T.H., Gurney G.G., Hughes T.P., Álvarez-Romero J.G., Day J.C., Grantham R., Grech A., Hoey A.S. (2019). Coral reef conservation in the Anthropocene: Confronting spatial mismatches and prioritizing functions. Biol. Conserv..

[42] Morais R.A., Depczynski M., Fulton C., Marnane M., Narvaez P., Huertas V., Brandl S.J., Bellwood D.R. (2020). Severe coral loss shifts energetic dynamics on a coral reef. Funct. Ecol..

[43] Crisp S.K., Tebbett S.B., Bellwood D.R. (2022). A critical evaluation of benthic phase shift studies on coral reefs. Mar. Environ. Res..

[44] Elma E., Gullström M., Yahya S.A.S., Jouffray J.-B., East H.K., Nyström M. (2023). Post-bleaching alterations in coral reef communities. Mar. Poll. Bull..

[45] Mills M.S., Ungermann M., Rigot G., den Haan J., Leon J.X., Schils T. (2024). Coral reefs in transition: Temporal photoquadrat analyses and validation of underwater hyperspectral imaging for resource-efficient monitoring in Guam. PLoS ONE.

[46] Kaly U.L., Jones G.P. (1994). Test of the effect of disturbance on ciguatera in Tuvalu. Mem. Qld. Mus..

[47] Lewis R.J., Sellin M., Gillespie N.C., Holmes M.J., Keys A., Street R., Smythe H., Thaggard H., Bryce S. (1994). Ciguatera and herbivores: Uptake and accumulation of ciguatoxins in *Ctenochaetus striatus* on the Great Barrier Reef. Mem. Qld. Mus..

[48] Diaz-Pulido G., McCook L.J. (2002). The fate of bleached corals: Patterns and dynamics of algal recruitment. Mar. Ecol. Prog. Ser..

[49] Doropoulos C., Roff G., Bozec Y.-M., Zupan M., Werminghausen J., Mumby P.J. (2016). Characterizing the ecological trade-offs throughout the early ontogeny of coral recruitment. Ecol. Monogr..

[50] Wolfe K., Kenyon T.M., Mumby P.J. (2021). The biology and ecology of coral rubble and implications for the future of coral reefs. Coral Reefs.

[51] Victoria-Salazar I., Ruiz-Zárate M.Á., Vega-Zepeda A., Bahena-Basave H. (2023). Benthic successional dynamics on settlement substrate in coral reefs lagoons. Mar. Biol..

[52] Tebbett S.B., Connolly S.R., Bellwood D.R. (2023). Benthic composition changes on coral reefs at global scales. Nat. Ecol. Evol..

[53] Richards Z.T., Haines L., Ross C., Preston S., Matthews T., Terriaca A., Black E., Lewis Y., Mannolini J., Dean P. (2024). Deoxygenation following coral spawning and low-level thermal stress trigger mass coral mortality at Coral Bay, Ningaloo Reef. Coral Reefs.

[54] Tester P.A., Kibler S.R., Holland W.C., Usup G., Vandersea M.W., Leaw C.P., Teen L.P., Larsen J., Mohammad-Noor N., Faust M.A. (2014). Sampling harmful benthic dinoflagellates: Comparison of artificial and natural substrate methods. Harmful Algae.

[55] Tester P.A., Litaker R.W., Soler-Onís E., Fernández-Zabala J., Berdalet E. (2022). Using artificial substrates to quantify *Gambierdiscus* and other toxic benthic dinoflagellates for monitoring purposes. Harmful Algae.

[56] Anderson D.M., Lobel P.S. (1987). The continuing enigma of ciguatera. Biol. Bull..

[57] Cruz-Rivera E., Villareal T.A. (2006). Macroalgal palatability and the flux of ciguatera toxins through marine food webs. Harmful Algae.

[58] Parsons M.L., Richlen M.L., Smith T.B., Solow A.R., Anderson D.M. (2021). Evaluation of 24-h screen deployments as a standardized platform to monitor *Gambierdiscus* populations in the Florida Keys and U.S. Virgin Islands. Harmful Algae.

[59] Bellwood D.R., Hughes T.P., Hoey A.S. (2006). Sleeping functional group drives coral-reef recovery. Curr. Biol..

[60] Ledlie M.H., Graham N.A.J., Bythell J.C., Wilson S.K., Jennings S., Polunin N.V.C., Hardcastle J. (2007). Phase shifts and the role of herbivory in the resilience of coral reefs. Coral Reefs.

[61] Morillo-Velarde P.S., Briones-Fourzán P., Álvarez-Filip L., Aguíñiga-García S., Sánchez-González A., Lozano-Álvarez E. (2018). Habitat degradation alters trophic pathways but not food chain length on shallow Caribbean coral reefs. Sci. Rep..

[62] Tebbett S.B., Bennett S., Bellwood D.R. (2023). A functional perspective on the meaning of the term ‘herbivore’: Patterns versus processes in coral reef fishes. Coral Reefs.

[63] Tebbett S.B., Crisp S.K., Evans R.C., Fulton C.J., Pessarrodona A., Wernberg T., Wilson S.K., Bellwood D.R. (2023). On the challenges of identifying benthic dominance on Anthropocene coral reefs. BioScience.

[64] Bellwood D.R., Brandl S.J., McWilliam M., Streit R.P., Yan H.F., Tebbett S.B. (2024). Studying functions on coral reefs: Past perspectives, current conundrums, and future potential. Coral Reefs.

[65] Edmunds P.J. (2024). Decadal-scale time series highlight the role of chronic disturbances in driving ecosystem collapse in the Anthropocene. Ecology.

[66] Parsons M.L., Settlemier C.J., Bienfang P.K. (2010). A simple model capable of simulating the population dynamics of *Gambierdiscus*, the benthic dinoflagellate responsible for ciguatera fish poisoning. Harmful Algae.

[67] Loeffler C.R., Richlen M.L., Brandt M.E., Smith T.B. (2015). Effects of grazing, nutrients, and depth on the ciguatera-causing dinoflagellate *Gambierdiscus* in the US Virgin Islands. Mar. Ecol. Prog. Ser..

[68] Kassim N.S., Lee L.K., Hii K.S., Azmi N.F.M., Baharudin S.N., Liu M., Gu H., Lim P.T., Leaw C.P. (2025). Molecular diversity of benthic harmful dinoflagellates on a tropical reef: Comparing natural and artificial substrate sampling methods using DNA metabarcoding and morphological analysis. Harmful Algae.

[69] Mangialajoa L., Fricke A., Perez-Gutierreza G., Catania D., Jauzeina C., Lemeea R. (2017). Benthic dinoflagellate integrator (BEDI): A new method for the quantification of benthic harmful algal blooms. Harmful Algae.

[70] Tebbett S.B., Emslie M.J., Jonker M.J., Ling S.D., Pratchett M.S., Siqueira A.C., Thompson A.A., Yan H.F., Bellwood D.R. (2025). Epilithic algal composition and the functioning of Anthropocene reefs. Mar. Pollut. Bull..

[71] Radford B., Puotinen M., Sahin D., Boutros N., Wyatt M., Gilmour J. (2024). A remote sensing model for coral recruitment habitat. Remote Sens. Environ..

[72] Waterhouse J., Schaffelke B., Bartley R., Eberhard R., Brodie J., Star M., Thorburn P., Rolfe J., Ronan M., Taylor B. (2017). Scientific Consensus Statement: Land Use Impacts on Great Barrier Reef Water Quality and Ecosystem Condition.

[73] Bozec Y.-M., Hock K., Mason R.A.B., Baird M.E., Castro-Sanguino C., Condie S.A., Puotinen M., Thompson A., Mumby P.J. (2022). Cumulative impacts across Australia’s Great Barrier Reef: A mechanistic evaluation. Ecol. Monogr..

[74] Kopecky K.L., Holbrook S.J., Partlow E., Cunningham M., Schmitt R.J. (2024). Changing disturbance regimes, material legacies, and stabilizing feedbacks: Dead coral skeletons impair key recovery processes following coral bleaching. Glob. Change Biol..

[75] Pratchett M.S., Hoey A.S., Wilson S.K., Messmer V., Graham N.A.J. (2011). Changes in biodiversity and functioning of reef fish assemblages following coral bleaching and coral loss. Diversity.

[76] Hahn S.T., Capra M.F. (1992). The cyanobacterium *Oscillatoria erythraea*—A potential source of the toxin in the ciguatera food-chain. Food Addit. Contam..

[77] Laurent D., Kerbrat A.-S., Darius H.T., Cirarad E., Golubic S., Benoit E., Sauviat M.-P., Chinain M., Molgo J., Pauillac S. (2008). Are cyanobacteria involved in ciguatera fish poisoning-like outbreaks in New Caledonia. Harmful Algae.

[78] Kerbrat A.-S., Darius H.T., Pauillac S., Chinain M., Laurent D. (2010). Detection of ciguatoxin-like and paralysing toxins in *Trichodesmium* spp. from New Caledonia lagoon. Mar. Pol. Bull..

[79] Yasumoto T., Nakajima I., Bagnis R., Adachi R. (1977). Finding of a dinoflagellate as a likely culprit for ciguatera. Bull. Jpn. Soc. Sci. Fish..

[80] Yasumoto T., Inoue A., Bagnis R., Garcon M. (1979). Ecological survey on a dinoflagellate possibly responsible for the induction of ciguatera. Bull. Jpn. Soc. Sci. Fish..

[81] Yasumoto T., Inoue A., Ochi T., Fujimoto K., Oshima Y., Fukuyo Y., Adachi R., Bagnis R. (1980). Environmental studies on a toxic dinoflagellate responsible for ciguatera. Bull. Jpn. Soc. Sci. Fish..

[82] Hurtel J.M., Chanteau A.U., Drollet J.H., Bagnis R. (1979). Culture en milieu artificiel du dinoflagelle responsable de la ciguatera. Rev. Int. Océanogr. Méd. Tome.

[83] Faust M.A. (1995). Observation of sand-dwelling toxic dinoflagellates (Dinophyceae) from widely differing sites, including two new species. J. Phycol..

[84] Nguyen-Ngoc L., Larsen J., Doan-Nhu H., Nguyen X.-V., Chomérat N., Lundholm N., Phan-Tan L., Viet Dao N., Nguyen N.-L., Nguyen H.-H. (2023). *Gambierdiscus* (gonyaulacales, dinophyceae) diversity in Vietnamese waters with description of *G. vietnamensis* sp. nov. J. Phycol..

[85] Chinain M., Gatti C.M., Roué M., Darius H.T., Subba Rao D.V. (2020). Ciguatera-Causing Dinoflagellates in the Genera *Gambierdiscus* and *Fukuyoa*: Distribution, Ecophysiology and Toxicology. Dinoflagellates.

[86] Chinain M., Gatti Howell C., Roué M., Ung A., Henry K., Revel T., Cruchet P., Viallon J., Darius H.T. (2023). Ciguatera poisoning in French Polynesia: A review of the distribution and toxicity of *Gambierdiscus* spp., and related impacts on food web components and human health. Harmful Algae.

[87] Murray J.S., Passfield E.M.F., Rhodes L.L., Puddick J., Finch S.C., Smith K.F., van Ginkel R., Mudge E.M., Nishimura T., Funaki H. (2024). Targeted metabolite fingerprints of thirteen *Gambierdiscus*, five *Coolia* and two *Fukuyoa* species. Mar. Drugs.

[88] Gillespie N.C., Lewis R.J., Burke J., Holmes M., Gabrie C., Salvat B. (1985). The Significance of the Absence of Ciguatoxin in a Wild Population of G. toxicus. Proceedings of the Fifth International Coral Reef Congress.

[89] Holmes M.J., Lewis R.J., Poli M.A., Gillespie N.C. (1991). Strain dependent production of ciguatoxin precursors (gambiertoxins) by *Gambierdiscus toxicus* (Dinophyceae) in culture. Toxicon.

[90] Chinain M., Faust M.A., Pauillac S. (1999). Morphology and molecular analyses of three toxic species of *Gambierdiscus* (Dinophyceae): *G. pacificus*, sp. nov., *G. australes*, sp. nov., and *G. polynesiensis*, sp. nov. J. Phycol..

[91] Anderson D.M., Alpermann T.J., Cembella A.D., Collos Y., Masseret E., Montresor M. (2012). The globally distributed genus *Alexandrium*: Multifaceted roles in marine ecosystems and impacts on human health. Harmful Algae.

[92] Clausing R.J., Annunziata C., Baker G., Lee C., Bittick S.J., Fong P. (2014). Effects of sediment depth on algal turf height are mediated by interactions with fish herbivory on a fringing reef. Mar. Ecol. Prog. Ser..

[93] Gordon S.E., Goatley C.H.R., Bellwood D.R. (2016). Low-quality sediments deter grazing by the parrotfish *Scarus rivulatus* on inner-shelf reefs. Coral Reefs.

[94] Tebbett S.B., Goatley C.H.R., Bellwood D.R. (2017). Fine sediments suppress detritivory on coral reefs. Mar. Poll. Bull..

[95] Tebbett S.B., Goatley C.H.R., Bellwood D.R. (2017). The effects of algal turf sediments and organic loads on feeding by coral reef surgeonfishes. PLoS ONE.

[96] Tebbett S.B., Goatley C.H.R., Bellwood D.R. (2018). Algal turf sediments across the Great Barrier Reef: Putting coastal reefs in perspective. Mar. Poll. Bull..

[97] Walker T.A. (1981). Dependence of phytoplankton chlorophyll on bottom resuspension in Cleveland Bay, Northern Queensland. Aust. J. Mar. Freshw. Res..

[98] Brodie J., Wolanski E., Lewis S., Bainbridge Z. (2012). An assessment of residence times of land-sourced contaminants in the Great Barrier Reef lagoon and the implications for management and reef recovery. Mar. Poll. Bull..

[99] Furnas M.J., Mitchell A.W. (1996). Nutrient inputs into the central Great Barrier Reef (Australia) from subsurface intrusions of Coral Sea waters: A two-dimensional displacement model. Cont. Shelf Res..

[100] Wooldridge S.A., Heron S.F., Brodie J.E., Done T.J., Masiri I., Hinrichs S. (2017). Excess seawater nutrients, enlarged algal symbiont densities and bleaching sensitive reef locations: 2. A regional-scale predictive model for the Great Barrier Reef, Australia. Mar. Poll. Bull..

[101] Baird M.E., Mongin M., Skerratt J., Margvelashvili N., Tickell S., Steven A.D.L., Robillot C., Ellis R., Waters D., Kaniewska P. (2021). Impact of catchment-derived nutrients and sediments on marine water quality on the Great Barrier Reef: An application of the eReefs marine modelling system. Mar. Poll. Bull..

[102] Shibuno T., Hashimoto K., Abe O., Takada Y. (1999). Short-term changes in the structure of a fish community following coral bleaching at Ishigaki Island, Japan. Galaxea.

[103] Cheal A.J., MacNeill M.A., Cripps E., Emslie M.J., Jonker M., Schaffelke B., Sweatman H. (2010). Coral-macroalgal phase shifts or reef resilience: Links with diversity and functional roles of herbivorous fishes on the Great Barrier Reef. Coral Reefs.

[104] Webley J., McInnes K., Teixeira D., Lawson A., Quinn R. (2015). Statewide Recreational Fishing Survey 2013–2014. Queensland Government Report. http://era.daf.qld.gov.au/id/eprint/6513/.

[105] Pessarrodona A., Filbee-Dexter K., Wernberg T. (2023). Recovery of algal turfs following removal. Mar. Environ. Res..

[106] Hughes T. (1994). Phase shifts, and large-scale degradation of a Caribbean coral reef. Science.

[107] Mumby P.J., Hastings A., Edwards H.J. (2007). Thresholds and the resilience of Caribbean coral reefs. Nature.

[108] Edwards C.B., Friedlander A.M., Green A.G., Hardt M.J., Sala E., Sweatman H.P., Williams I.D., Zgliczynski B., Sandin S.A., Smith J.E. (2014). Global assessment of the status of coral reef herbivorous fishes: Evidence for fishing effects. Proc. R. Soc. B.

[109] Arias-González J.E., Fung T., Seymour R.M., Garza-Pérez J.R., Acosta-González G., Bozec Y.-M., Johnson C.R. (2017). A coral-algal phase shift in Mesoamerica not driven by changes in herbivorous fish abundance. PLoS ONE.

[110] Bruno J.F., Sweatman H., Precht W.F., Selig E.R., Schutte V.G.W. (2009). Assessing evidence of phase shifts from coral to macroalgal dominance on coral reefs. Ecology.

[111] Adam T.C., Schmitt R.J., Holbrook S.J., Brooks A.J., Edmunds P.J., Carpenter R.C., Bernardi G. (2011). Herbivory, connectivity, and ecosystem resilience: Response of a coral reef to a large-scale perturbation. PLoS ONE.

[112] Menge B.A., Gravem S.A., Richmond E., Noble M.M. (2023). A unified meta-ecosystem dynamics model: Integrating herbivore-plant subwebs with the intermittent upwelling hypothesis. Ecosphere.

[113] Bittick S.J., Fong C.R., Clausing R.J., Harvey J.D., Johnson T.M., Frymann T.A., Fong P. (2020). Herbivory strength is similar or even greater in algal- compared to coral-dominated habitats on a recovering coral reef. Mar. Ecol. Prog. Ser..

[114] Adam T.C., Holbrook S.J., Burkepile D.E., Speare K.E., Brooks A.J., Ladd M.C., Shantz A.A., Thurber R.V., Schmitt R.J. (2022). Priority effects in coral–macroalgae interactions can drive alternate community paths in the absence of top-down control. Ecology.

[115] Cline T.J., Allgeier J.E. (2022). Fish community structure and dynamics are insufficient to mediate coral resilience. Nat. Ecol. Evol..

[116] Cook D.T., Schmitt R.J., Holbrook S.J., Moeller H.V. (2024). Modeling the effects of selectively fishing key functional groups of herbivores on coral resilience. Ecosphere.

[117] Randazzo-Eisemann Á., Molina-Hernández A.L., Alvarez-Filip L., Garza-Pérez J.R. (2024). Strong linkage between parrotfish functions and habitat characteristics. PLoS ONE.

[118] Xu C., Chen W., Hu J. (2024). Deterministic and stochastic analysis of a coral reef ecosystem with grazed macroalgae. Int. J. Biomath..

[119] Sperr A.E., Doucette G.J., Yasumoto T., Oshima Y., Fukuyo Y. (1996). Variation in growth rate and ciguatera toxin production among geographically distinct isolates of Gambierdiscus toxicus. Harmful and Toxic Algal Blooms.

[120] Lartigue J., Jester E.L.E., Dickey R.W., Villareal T.A. (2009). Nitrogen source effects on the growth and toxicity of two strains of the ciguatera-causing dinoflagellate *Gambierdiscus toxicus*. Harmful Algae.

[121] Litaker R.W., Holland W.C., Hardison D.H., Pisapia F., Hess P., Kibler S.R., Tester P.A. (2017). Ciguatoxicity of *Gambierdiscus* and *Fukuyoa* species from the Caribbean and Gulf of Mexico. PLoS ONE.

[122] Vacarizas J., Benico G., Austero N., Azanza R. (2018). Taxonomy and toxin production of *Gambierdiscus carpenteri* (Dinophyceae) in a tropical marine ecosystem: The first record from the Philippines. Mar. Pol. Bull..

[123] Chinain M., Darius H.T., Ung A., Cruchet P., Wang Z., Ponton D., Laurent D., Pauillac S. (2010). Growth and toxin production in the ciguatera-causing dinoflagellate *Gambierdiscus polynesiensis* (Dinophyceae) in culture. Toxicon.

[124] Darius H.T., Revel T., Viallon J., Sibat M., Cruchet P., Longo S., Hardison D.R., Holland W.C., Tester P.A., Litaker R.W. (2022). Comparative study on the performance of three detection methods for the quantification of Pacific ciguatoxins in French Polynesian strains of *Gambierdiscus polynesiensis*. Toxins.

[125] Villareal T.A., Morton S.L. (2002). Use of cell-specific PAM-fluorometry to characterize host shading in the epiphytic dinoflagellate *Gambierdiscus toxicus*. Mar. Ecol..

[126] Tebbett S.B., Bellwood D.R., Bassett T., Cuttler M.V.W., Moustaka M., Wilson S.K., Yan H.F., Evans R.D. (2024). The limited role of herbivorous fishes and turf-based trophic pathways in the functioning of turbid coral reefs. Rev. Fish Biol. Fish..

[127] Darius H.T., Revel T., Cruchet P., Viallon J., Gatti C.M.I., Sibat M., Hess P., Chinain M. (2021). Deep-water fish are potential vectors of ciguatera poisoning in the Gambier Islands, French Polynesia. Mar. Drugs.

[128] Wang B., Yao M., Zhou J., Tan S., Jin H., Zhang F., Mal Y.L., Wu J., Chan L.L., Cai Z. (2018). Growth and toxin production of *Gambierdiscus* spp. can be regulated by quorum-sensing bacteria. Toxins.

[129] Price D.C., Farinholt N., Gates C., Shumaker A., Wagner N.E., Bienfang P., Bhattacharya D. (2016). Analysis of *Gambierdiscus* transcriptome data supports ancient origins of mixotrophic pathways in dinoflagellates. Environ. Microbiol..

[130] Faust M.A., Reguera B., Blanco J., Fernández M.L., Wyatt T. (1998). Mixotrophy in tropical benthic dinoflagellates. Harmful Algae, Proceedings of the VIII International Conference on Harmful Algae, Vigo, Spain, 25–29 June 1997.

[131] Selander E., Thor P., Toth G., Pavia H. (2006). Copepods induce paralytic shellfish toxin production in marine dinoflagellates. Proc. R. Soc. B.

[132] Bergkvist J., Selander E., Pavia H. (2008). Induction of toxin production in dinoflagellates: The grazer makes a difference. Oecologia.

[133] Tammilehto A., Nielsen T.G., Krock B., Møller E.F., Lundholm N. (2015). Induction of domoic acid production in the toxic diatom *Pseudo-nitzschia seriata* by calanoid copepods. Aquat. Toxicol..

[134] Senft-Batoh C.D., Dam H.G., Shumway S.E., Wikfors G.H., Schlichting C.D. (2015). Influence of predator–prey evolutionary history, chemical alarm-cues, and feeding selection on induction of toxin production in a marine dinoflagellate. Limnol. Oceanogr..

[135] Lundholm N., Krock B., John U., Skov J., Cheng J., Pančić M., Wohlrab S., Rigby K., Nielsen T.G., Selander E. (2018). Induction of domoic acid production in diatoms—Types of grazers and diatoms are important. Harmful Algae.

[136] Park G., Norton L., Avery D., Dam H.G. (2023). Grazers modify the dinoflagellate relationship between toxin production and cell growth. Harmful Algae.

[137] Zhang S., Zheng T., Zhou M., Niu B., Li Y. (2024). Exposure to the mixotrophic dinoflagellate *Lepidodinium* sp. and its cues increase toxin production of *Pseudo-nitzschia multiseries*. Sci. Total Environ..

[138] Karban R. (2011). The ecology and evolution of induced resistance against herbivores. Funct. Ecol..

[139] Gillespie N.C., Holmes M.J., Burke J.B., Doley J., Anderson D.M., White A.W., Baden D.G. (1985). Distribution and periodicity of *Gambierdiscus toxicus* in Queensland, Australia. Toxic Dinoflagellates.

[140] Holmes M., Lewis R.J., Sellin M., Street R. (1994). The origin of ciguatera in Platypus Bay, Australia. Mem. Qld Mus..

[141] Legrand A.-M., Reguera B., Blanco J., Fernández M.L., Wyatt T. (1998). Ciguatera toxins: Origin, transfer through the food chain and toxicity to humans. Harmful Algae.

[142] Longo S., Sibat M., Viallon J., Darius H.T., Hess P., Chinain M. (2019). Intraspecific variability in the toxin production and toxin profiles of in vitro cultures of *Gambierdiscus polynesiensis* (Dinophyceae) from French Polynesia. Toxins.

[143] Richlen M.L., Horn K., Uva V., Fachon E., Heidmann S.L., Smith T.B., Parsons M.L., Anderson D.M. (2024). *Gambierdiscus* species diversity and community structure in St. Thomas, USVI and the Florida Keys, USA. Harmful Algae.

[144] Rhodes L.L., Smith K.F., Murray J.S., Nishimura T., Finch S.C. (2020). Ciguatera fish poisoning: The risk from an Aotearoa/New Zealand perspective. Toxins.

[145] Fraga S., Rodríguez F., Caillaud A., Diogène J., Raho N., Zapata M. (2011). *Gambierdiscus excentricus* sp. nov. (Dinophyceae), a benthic toxic dinoflagellate from the Canary Islands (NE Atlantic Ocean). Harmful Algae.

[146] Pisapia F., Holland W.C., Hardison D.R., Litaker R.W., Fraga S., Nishimura T., Adachi M., Nguyen-Ngoc L., Sécheta V., Amzil Z. (2017). Toxicity screening of 13 *Gambierdiscus* strains using neuro-2a and erythrocyte lysis bioassays. Harmful Algae.

[147] Robertson A., Richlen M.L., Erdner D., Smith T.B., Anderson D.M., Liefer J.D., Xu Y., McCarron P., Miles C.O., Parsons M.L., Hess P. (2018). Toxicity, chemistry, and implications of *Gamberdiscus silvae*: A ciguatoxin superbug in the Greater Caribbean Region. 18th International Conference for Harmful Algae [Abstract Book].

[148] Rossignoli A.E., Tudó A., Bravo I., Díaz P.A., Diogène J., Riobó P. (2020). Toxicity characterisation of *Gambierdiscus* species from the Canary Islands. Toxins.

[149] Gaiani G., Leonardo S., Tudó À., Toldrà A., Rey M., Andree K.B., Tsumuraya T., Hirama M., Diogène J., O’Sullivan C.K. (2020). Rapid detection of ciguatoxins in *Gambierdiscus* and Fukuyoa with immunosensing tools. Ecotoxicol. Environ. Saf..

[150] Chateau-Degat M.-L., Chinain M., Cerf N., Gingras S., Hubert B., Dewailly É. (2005). Seawater temperature, *Gambierdiscus* spp. variability and incidence of ciguatera in French Polynesia. Harmful Algae.

[151] Liefer J.D., Richlen M.L., Smith T.B., DeBose J.L., Xu Y., Anderson D.M., Robertson A. (2021). Asynchrony of *Gambierdiscus* spp. abundance and toxicity in the U.S. Virgin Islands: Implications for monitoring and management of ciguatera. Toxins.

[152] Litaker R.W., Vandersea M.W., Faust M.A., Kibler S.R., Nau A.W., Holland W.C., Chinain M., Holmes M.J., Tester P.A. (2010). Global distribution of ciguatera causing dinoflagellates in the genus *Gambierdiscus*. Toxicon.

[153] Kingsford M.J. (1992). Spatial and temporal variation in predation on reef fishes by coral trout (*Plectropomus leopardus*, Serranidae). Coral Reefs.

[154] St John J., Russ G.R., Brown I.W., Squire L.C. (2001). The diet of the large coral reef serranid *Plectropomus leopardus* in two fishing zones on the Great Barrier Reef, Australia. Fish. Bull..

[155] Matley J.K., Maes G.E., Devloo-Delva F., Huerlimann R., Chua G., Tobin A.J., Fisk A.T., Simpfendorfer C.A., Heupel M.R. (2018). Integrating complementary methods to improve diet analysis in fishery-targeted species. Ecol. Evol..

[156] Magnelia S.J., Kohler C.C., Tindall D.R. (1992). Acanthurids do not avoid consuming cultured toxic dinoflagellates yet do not become ciguatoxic. Trans. Am. Fish. Soc..

[157] Clausing R.J., Losen B., Oberhaensli F.R., Darius H.T., Sibat M., Hess P., Swarzenski P.W., Chinain M., Bottein M.-Y.D. (2018). Experimental evidence of dietary ciguatoxin accumulation in an herbivorous coral reef fish. Aquat. Toxicol..

[158] Darius H.T., Ponton D., Revel T., Cruchet P., Ung A., Tchou Fouc M., Chinain M. (2007). Ciguatera risk assessment in two toxic sites of French Polynesia using the receptor-binding assay. Toxicon.

[159] FishBase World Wide Web Electronic Publication, Version (02/2024). Froese, R.; Pauly, D., Eds.; 2024. https://www.fishbase.se/search.php.

[160] Bray D.J., Bray D.J., Gomon M.F. (2018). Introduction to Australia’s Fishes. Fishes of Australia.

[161] Darias-Dágfeel Y., Sanchez-Henao A., Padilla D., Martín M.V., Ramos-Sosa M.J., Poquet P., Barreto M., Sergent F.S., Jerez S., Real F. (2024). Effects on biochemical parameters and animal welfare of dusky grouper (*Epinephelus marginatus*, Lowe 1834) by feeding CTX toxic flesh. Animals.

[162] Leigh G.M., Campbell A.B., Lunow C.P., O’Neill M.F. (2014). Stock Assessment of the Queensland East Coast Common Coral Trout (Plectropomus leopardus) Fishery.

[163] Fenner P.J., Lewis R.J., Williamson J.A., Williams M.L. (1997). A Queensland family with ciguatera after eating coral trout. Med. J. Aust..

[164] Greenwood N.D.W., Sweeting C.J., Polunin N.V.C. (2010). Elucidating the trophodynamics of four coral reef fishes of the Solomon Islands using δ15N and δ13C. Coral Reefs.

[165] Bierwagen S.L., Pethybridge H., Heupel M.R., Chin A., Simpfendorfer C.A. (2019). Trophic niches determined from fatty acid profiles of sympatric coral reef mesopredators. Mar. Ecol. Prog. Ser..

[166] Skinner C., Newman S.P., Mill A.C., Newton J., Polunin N.V.C. (2019). Prevalence of pelagic dependence among coral reef predators across an atoll seascape. J. Anim. Ecol..

[167] Frisch A.J., Ireland M., Baker R. (2014). Trophic ecology of large predatory reef fishes: Energy pathways, trophic level, and implications for fisheries in a changing climate. Mar. Biol..

[168] Emslie M.J., Cheal A.J., Logan M. (2017). The distribution and abundance of reef-associated predatory fishes on the Great Barrier Reef. Coral Reefs.

[169] Frisch A., van Herwerden L. (2006). Field and experimental studies of hybridization between coral trouts, *Plectropomus leopardus* and *Plectropomus maculatus* (Serranidae), on the Great Barrier Reef, Australia. J. Fish Biol..

[170] Van Noord J.E., Lewallen E.A., Pitman R.L. (2013). Flyingfish feeding ecology in the eastern Pacific: Prey partitioning within a speciose epipelagic community. J. Fish Biol..

[171] Zhu J., Li J., Wu J., Liu X., Lin Y., Deng H., Qin X., Wong M.H., Chan L.L. (2024). The prevalence of marine lipophilic phycotoxins causes potential risks in a tropical small island developing State. Environ. Sci. Technol..

[172] Skinner C., Gallimore S., Polunin N.V.C., Rushton S., Newman S.P., Desbiens A.A., Mill A.C. (2024). Corresponding planktivore and predator spatial distributions in an oceanic coral reef system. Coral Reefs.

[173] Hempson T.N., Graham N.A.J., MacNeil M.A., Williamson D.W., Jones G.P., Almany G.R. (2017). Coral reef mesopredators switch prey, shortening food chains, in response to habitat degradation. Ecol. Evol..

[174] Hempson T.N., Graham N.A.J., MacNeil M.A., Bodin N., Wilson S.K. (2018). Regime shifts shorten food chains for mesopredators with potential sublethal effects. Funct. Ecol..

[175] Wilson S.K., Graham N.A.J., Pratchett M.S., Jones G.P., Polunin N.V.C. (2006). Multiple disturbances and the global degradation of coral reefs: Are reef fishes at risk or resilient?. Glob. Change Biol..

[176] Graham N.A.J., Wilson S.K., Jennings S., Polunin N.V.C., Robinson J., Bijoux J.P., Daw T.M. (2007). Lag effects in the impacts of mass coral bleaching on coral reef fish, fisheries, and ecosystems. Conserv. Biol..

[177] Huang M., Wei S., Li Q., Gao K., Peng Z., Chen Y., Zhou W., Wei F. (2023). Degradation of coral reefs altered the community trophic structure and reduced the shoaling size of fish. Front. Conserv. Sci..

[178] Halstead B.W., Bunker N.C. (1954). A survey of the poisonous fishes of the Phoenix Islands. Copeia.

[179] Halstead B.W. (1988). Poisonous and Venomous Marine Animals of the World.

[180] Robertson D.R., Van Tassell J. (2023). Shorefishes of the Greater Caribbean: Online Information System, Version 3.0.

[181] Díaz-Ascenio L., Clausing R.J., Vandersea M., Chamero-Lago D., Gómez-Batista M., Hernández-Albernas J.I., Chomérat N., Rojas-Abrahantes G., Litaker R.W., Tester P. (2019). Ciguatoxin occurrence in food-web components of a Cuban coral reef ecosystem: Risk-assessment implications. Toxins.

[182] Roff G., Mumby P.J. (2012). Global disparity in the resilience of coral reefs. Trends Ecol. Evol..

[183] Bellwood D.R., Hughes T.P., Folke C., Nyström M. (2004). Confronting the coral reef crisis. Nature.

[184] Hemingson C.R., Bellwood D.R. (2020). Greater multihabitat use in Caribbean fishes when compared to their Great Barrier Reef counterparts. Est. Coast. Shelf Sci..

[185] Siqueira A.C., Bellwood D.R., Cowman P.F. (2019). The evolution of traits and functions in herbivorous coral reef fishes through space and time. Proc. R. Soc. B.

[186] Siqueira A.C., Bellwood D.R., Cowman P.F. (2019). Historical biogeography of herbivorous coral reef fishes: The formation of an Atlantic fauna. J. Biogeogr..

[187] O’Dea A., Lessios H.A., Coates A.G., Eytan R.I., Restrepo-Moreno S.A., Cione A.L., Collins L.S., de Queiroz A., Farris D.W., Norris R.D. (2016). Formation of the isthmus of Panama. Sci. Adv..

[188] Carlson A.E. (2011). Ice sheets and sea level in Earth’s past. Nat. Educ. Knowl..

[189] Voris H.K. (2000). Maps of Pleistocene Sea levels in southeast Asia: Shorelines, river systems and time durations. J. Biogeogr..

[190] Larsson M.E., Laczka O.F., Suthers I.M., Ajani P.A., Doblin M.A. (2018). Hitchhiking in the East Australian current: Rafting as a dispersal mechanism for harmful epibenthic dinoflagellates. Mar. Ecol. Prog. Ser..

[191] Saura S., Bodin Ö., Fortin M.J. (2014). Stepping stones are crucial for species’ long-distance dispersal and range expansion through habitat networks. J. Appl. Ecol..

[192] Loeffler C.R., Spielmeyer A., Blaschke V., Bodi D., Kappenstein O. (2023). Ciguatera poisoning in Europe: A traceback to Indian Ocean sourced snapper fish (*Lutjanus bohar*). Food Control.

[193] Li X., Lew K., Leyau Y.L., Shen P., Chua J., Lin K.J., Wu Y., Chan S.H. (2025). Application of high-resolution mass spectrometry for ciguatoxin detection in fish from the Asia–Pacific Region. Toxins.

[194] Feng M., Zhang N., Wijffles S. (2018). The Indonesian throughflow, its variability and centennial change. Geosci. Lett..

[195] Cao Z., Li M., Gordon A.L., Wang D. (2025). Enhanced Indonesian Throughflow heat transport prolongs the recharge process during triple La Niña events. Environ. Res. Lett..

[196] Habibi N., Uddin S., Bottein M.-Y.D., Faizuddin M. (2021). Ciguatera in the Indian Ocean with special insights on the Arabian Sea and adjacent gulf and seas: A review. Toxins.

[197] Lucas R.E., Lewis R.J., Taylor J.M. (1997). Pacific ciguatoxin-1 associated with a large common-source outbreak of ciguatera in East Arnhem Land, Australia. Nat. Toxins.

[198] Post D.M. (2002). Using stable isotopes to estimate trophic position: Models, methods, and assumptions. Ecology.

[199] Tebbett S.B., Goatley C.H.R., Bellwood D.R. (2017). Clarifying functional roles: Algal removal by the surgeonfishes *Ctenochaetus striatus* and *Acanthurus nigrofuscus*. Coral Reefs.

[200] Tebbett S.B., Goatley C.H.R., Huertas V., Mihalitsis M., Bellwood D.R. (2018). A functional evaluation of feeding in the surgeonfish *Ctenochaetus striatus*: The role of soft tissues. R. Soc. Open Sci..

[201] Thompson C., Silva R., Gibran F.Z., Bacha L., de Freitas M.A.M., Thompson M., Landuci F., Tschoeke D., Zhang X.-H., Wang X. (2024). The Abrolhos nominally herbivorous coral reef fish *Acanthurus chirurgus*, *Kyphosus* sp., *Scarus trispinosus*, and *Sparisoma axillare* have similarities in feeding but species-specific microbiomes. Microb. Ecol..

[202] Ledreux A., Brand H., Chinain M., Bottein M.-Y.D. (2014). Dynamics of ciguatoxins from *Gambierdiscus polynesiensis* in the benthic herbivore *Mugil cephalus*: Trophic transfer implications. Harmful Algae.

[203] Li J., Mak Y.L., Chang Y.-H., Xiao C., Chen Y.-M., Shen J., Wang Q., Ruan Y., Lam P.K.S. (2020). Uptake and depuration kinetics of Pacific ciguatoxins in orange-spotted grouper (*Epinephelus coioides*). Environ. Sci. Technol..

[204] Clements K.D., German D.P., Piché J., Tribollet A., Choat J.H. (2017). Integrating ecological roles and trophic diversification on coral reefs: Multiple lines of evidence identify parrotfishes as microphages. Biol. J. Linn. Soc..

[205] Nicholson G.M., Clments K.D. (2020). Resolving resource partitioning in parrotfishes (Scarini) using microhistology of feeding substrata. Coral Reefs.

[206] Russ G.R., Questel S.-L.A., Rizzari J.R., Alcala A.C. (2015). The parrotfish-coral relationship: Refuting the ubiquity of a prevailing paradigm. Mar. Biol..

[207] Vallès H., Oxenford H.A. (2014). Parrotfish Size: A simple yet useful alternative indicator of fishing effects on Caribbean Reefs?. PLoS ONE.

[208] Bozec Y.-M., O’Farrell S., Bruggemannd J.H., Luckhurstf B.E., Mumby P.J. (2016). Tradeoffs between fisheries harvest and the resilience of coral reefs. Proc. Natl. Acad. Sci. USA.

[209] Boucaud-Maitre D., Vernoux J.-P., Pelczar S., Daudens-Vaysse E., Aubert L., Boa S., Ferracci S., Garnier R. (2018). Incidence and clinical characteristics of ciguatera fish poisoning in Guadeloupe (French West Indies) between 2013 and 2016: A retrospective cases-series. Sci. Rep..

[210] Tester P.A., Feldman R.L., Nau A.W., Kibler S.R., Litaker R.W. (2010). Ciguatera fish poisoning and sea surface temperatures in the Caribbean Sea and the West Indies. Toxicon.

[211] Kindinger T.L., Adam T.C., Baum J.K., Dimoff S.A., Hoey A.S., Williams I.D. (2024). Herbivory through the lens of ecological processes across Pacific coral reefs. Ecosphere.

[212] Robinson J.P.W., McDevitt-Irwin J.M., Dajka J.-C., Hadj-Hammou J., Howlett S., Graba-Landry A., Hoey A.S., Nash K.L., Wilson S.K., Graham N.A.J. (2020). Habitat and fishing control grazing potential on coral reefs. Funct. Ecol..

[213] Munsterman K.S., Allgeier J.E., Peters J.R., Burkepile D.E. (2021). A view from both ends: Shifts in herbivore assemblages impact top-down and bottom-up processes on coral reefs. Ecosystems.

[214] DeMartini E.E., Smith J., Mora C. (2015). Effects of fishing on the fishes and habitat of coral reefs. Ecology of Fishes on Coral Reefs.

[215] Houk P., Cuetos-Bueno J., Kerr A.M., McCann K. (2018). Linking fishing pressure with ecosystem thresholds and food web stability on coral reefs. Ecol. Monogr..

[216] Kuempel C.D., Altieri A.H. (2017). The emergent role of small-bodied herbivores in pre-empting phase shifts on degraded coral reefs. Sci. Rep..

[217] Heenan A., Hoey A.S., Williams G.J., Williams I.D. (2016). Natural bounds on herbivorous coral reef fishes. Proc. R. Soc. B.

[218] Jones H.P., Schmitz O.J. (2023). Rapid recovery of damaged ecosystems. PLoS ONE.

[219] Morais J., Tebbett S.B., Morais R.A., Bellwood D.R. (2023). Natural recovery of corals after severe disturbance. Ecol. Lett..

[220] NOAA 2024 NOAA Confirms 4th Global Coral Bleaching Event. https://www.noaa.gov/news-release/noaa-confirms-4th-global-coral-bleaching-event.

